# The Effect of Preoperative Use of High- vs. Low-PAP-Inducing-Potential FP Agonists on the Surgical Outcomes of Trabeculectomy and AGV Implantation

**DOI:** 10.3390/jcm14196940

**Published:** 2025-09-30

**Authors:** Iyo Yamazaki, Masayo Kimura, Risaki Sakamoto, Yukiko Kawai, Tomomi Tsukamura, Hiroshi Morita, Aki Kato, Hironori Ozeki, Miho Nozaki, Tsutomu Yasukawa

**Affiliations:** 1 Department of Ophthalmology and Visual Science, Nagoya City University Graduate School of Medical Sciences, Nagoya 467-8601, Aichi, Japan; 14ymym.17@gmail.com (I.Y.); risakit13@gmail.com (R.S.); ykawai.medical@gmail.com (Y.K.); tomo.jk0629@gmail.com (T.T.); hiroshi1966morita@au.com (H.M.); akikato@cjimc-hp.jp (A.K.); miho.nozaki@gmail.com (M.N.); yasukawa@med.nagoya-cu.ac.jp (T.Y.); 2 Ozeki Eye Clinic, Ama-Gun 497-0050, Aichi, Japan; ozeki@dc5.so-net.ne.jp

**Keywords:** glaucoma, trabeculectomy, Ahmed glaucoma valve implantation, prostanoid FP receptor agonist, prostaglandin-associated periorbitopathy

## Abstract

**Background**: Prostanoid FP receptor agonists (FP agonists) are widely used as first-line therapies for glaucoma but differ in their potential to induce prostaglandin-associated periorbitopathy (PAP), which may affect surgical outcomes. While several studies have reported an association between PAP and trabeculectomy failure, the impact of these agents on tube shunt procedures such as Ahmed glaucoma valve (AGV) implantation is not well established. **Methods**: We retrospectively analyzed 298 eyes of 221 patients who underwent trabeculectomy (*n* = 162) or AGV implantation (*n* = 136) between 2018 and 2023. The eyes were stratified by preoperative FP agonist use into the high-PAP-inducing-potential (bimatoprost or travoprost) and low-PAP-inducing-potential (latanoprost or tafluprost) groups. The primary outcome was the cumulative 2-year surgical survival rate under three intraocular pressure (IOP)-based definitions. **Results**: In the trabeculectomy group, the high-PAP-potential group had significantly lower 2-year survival rates than the low-PAP-potential group under all definitions. Cox proportional hazards analysis identified use of a high-PAP-potential FP agonist as a significant risk factor for surgical failure. In the AGV group, a difference between groups was seen only under the most lenient definition, with no differences under stricter criteria. **Conclusions**: The preoperative use of high-PAP-potential FP agonists is associated with poorer outcomes after trabeculectomy. Although the effect on AGV implantation appears limited, it may still influence early postoperative results. These findings underscore the need to consider PAP risk and medication history when selecting surgical procedures for glaucoma.

## 1. Introduction

Prostanoid FP receptor agonists (FP agonists), including bimatoprost, latanoprost, tafluprost, and travoprost, are widely used as first-line medications for glaucoma. However, these agents are known to induce various periorbital tissue changes collectively referred to as prostaglandin-associated periorbitopathy (PAP), including deepening of the upper-eyelid sulcus (DUES), ptosis, enophthalmos, and dermatochalasis [1]. Experimental studies have shown that these changes result from FP-receptor-mediated inhibition of adipogenesis and increased expression of type I collagen in orbital tissues, leading to periorbital tissue remodeling [2,3]. The incidence of PAP varies considerably depending on the specific agent. Bimatoprost has been associated with a high incidence of PAP (approximately 60–90%) [4,5,6], followed by travoprost (50–70%) [5,7], whereas latanoprost (6–41%) [5,6,8,9,10] and tafluprost (14–19%) [5,11,12] are associated with lower rates. These structural changes are thought to affect surgical outcomes, particularly in filtering procedures such as trabeculectomy, where successful bleb formation and maintenance are critical. For example, Miki et al. reported reduced trabeculectomy survival in regard to eyes treated with bimatoprost [13]. Moreover, the presence of DUES—more frequent with bimatoprost and travoprost—has been associated with poorer surgical outcomes [13]. A recent study using the Shimane University PAP (SU–PAP) Grading System further demonstrated that higher PAP severity is associated with lower surgical success [14].

In cases refractory to maximally tolerated glaucoma medications and conventional surgical interventions including trabeculectomy, tube shunt procedures are often considered [15]. Among these, Ahmed glaucoma valve (AGV) implantation is widely used, and its efficacy has been demonstrated in large clinical trials such as the Tube Versus Trabeculectomy (TVT) and Primary Tube Versus Trabeculectomy (PTVT) studies [16,17]. AGV implantation involves the insertion of a drainage tube and the creation of a bleb located approximately 8–10 mm posterior to the limbus. Due to its distinct anatomical configuration and different mechanism of aqueous outflow, AGV may be less affected by the structural changes associated with PAP. In fact, a recent study reported no significant associations between the severity of PAP and the surgical success rate following AGV implantation [18].

Given the anatomical and functional differences between trabeculectomy and AGV implantation, we aimed to investigate whether the preoperative use of FP agonists—particularly those with a high potential to induce PAP and DUES—differentially affects surgical outcomes. Based on previous reports [4,5,6,7,8,9,10,11,12,13], patients were stratified into two groups according to the agents used: the high-PAP/DUES-inducing-potential group (bimatoprost or travoprost) and the low-PAP/DUES-inducing potential group (latanoprost or tafluprost). Two-year outcomes were then analyzed separately for each procedure.

## 2. Materials and Methods

### 2.1. Study Design and Ethics

In this retrospective study, we reviewed the medical records of patients who underwent trabeculectomy and/or AGV implantation at Nagoya City University Hospital between January 2018 and September 2023. Only those with a postoperative follow-up period of at least one year were included in this study. The study was approved by the Ethics Committee of Nagoya City University (approval number: 60-23-0057) and registered with the UMIN Clinical Trials Registry (UMIN000057374).

### 2.2. Patients and Subgroups

The FP agonists used preoperatively included bimatoprost (0.03%) (Lumigan^®^ Ophthalmic Solution; Senju Pharmaceutical Co., Ltd., Osaka, Japan), latanoprost (0.005%) (Xalatan^®^ Eye Drops; Pfizer Inc., Tokyo, Japan), tafluprost (0.0015%) (Tapros^®^ Ophthalmic Solution; Santen Pharmaceutical Co., Ltd., Osaka, Japan), and travoprost (0.004%) (Travatan Z^®^ Ophthalmic Solution; Alcon Japan, Ltd., Tokyo, Japan). Eyes were categorized into two groups according to the type of FP agonist used: the high-PAP-potential group (bimatoprost or travoprost) and the low-PAP-potential group (latanoprost or tafluprost). Eyes were classified according to the type of FP agonist administered immediately prior to surgery, regardless of prior changes in medication or duration of use.

Trabeculectomy was performed using a standardized technique, which included a fornix-based conjunctival and Tenon’s capsule incision; the creation of a 3.5 × 3.5 mm single- or double-layer scleral flap at the surgeon’s discretion; the application of mitomycin C (0.4 mg/mL) for 3 min, followed by thorough irrigation with balanced salt solution; excision of the trabecular meshwork; peripheral iridectomy; and closure of the scleral flap and conjunctiva with 10-0 nylon sutures. Postoperative laser suture lysis was performed when necessary.

AGV implantation was performed using the Ahmed FP7 Glaucoma Valve (New World Medical, Rancho Cucamonga, CA, USA) in all cases. The procedure involved dissection of the conjunctiva and Tenon’s capsule to accommodate the device, fixation of the endplate to the sclera 8–10 mm posterior to the limbus, the insertion of a tube into either the ciliary sulcus or vitreous cavity at the surgeon’s discretion, coverage of the limbal portion of the tube with either a donor scleral patch or a half-thickness scleral flap, and closure of the conjunctiva.

### 2.3. Outcome Measurements

Outcome measures included intraocular pressure (IOP), use of anti-glaucomatous medications, and surgical survival rates. Surgical failure was defined as reoperation for glaucoma or loss of light perception. Reoperation included any additional glaucoma surgery (e.g., repeat trabeculectomy or tube shunt implantation) or laser procedures such as selective laser trabeculoplasty (SLT), conventional transscleral cyclophotocoagulation (CPC), and micropulse laser CPC (MP-CPC). Postoperative procedures such as bleb needling, laser suture lysis, or Yttrium–Aluminum–Garnet (YAG) laser use for relieving tube occlusion were not considered reoperations.

Surgical failure was defined based on IOP control, using three increasingly stringent criteria derived from the TVT and PTVT studies:

- Definition A: IOP > 21 mmHg or <20% reduction from baseline;
- Definition B: IOP > 17 mmHg or <20% reduction from baseline;
- Definition C: IOP > 14 mmHg.

All thresholds had to be met on two consecutive follow-up visits after 3 months. Eyes that required glaucoma reoperation or experienced loss of light perception were considered surgical failures regardless of IOP.

The primary outcome was the cumulative surgical survival rates stratified by the PAP-inducing potential of FP agonists used preoperatively: the high-PAP-potential group (bimatoprost or travoprost) versus the low-PAP-potential group (latanoprost or tafluprost).

IOP was measured using either a Goldmann applanation tonometer or an iCare rebound tonometer (iCare; Icare Finland Oy, Vantaa, Finland). IOP was evaluated at 3, 6, 12, 18, and 24 months postoperatively.

The anti-glaucomatous medication score was calculated as follows: 1 point per topical agent, 2 points for fixed-combination drops, and 1 point for oral carbonic anhydrase inhibitors, regardless of dosage.

### 2.4. Statistical Analysis

Statistical analyses were conducted using IBM SPSS Statistics version 30. Univariate comparisons were performed using the Mann–Whitney U test for continuous variables, including age, IOP, and medication score, and Pearson’s χ^2^ test for categorical variables, including gender, glaucoma type, history of prior glaucoma surgery, lens status (phakic or pseudophakic/intraocular lens), and preoperative FP agonist use. The Friedman test was used to analyze variance for nonparametric data with three or more time points, while the Wilcoxon signed-rank test was used for paired comparisons between two time points. Generalized estimating equations (GEEs) were used to analyze longitudinal data. Cumulative survival rates were calculated using the Kaplan–Meier method and compared using the log-rank test. Kaplan–Meier survival analyses were performed separately for Definitions A–C. Factors associated with the survival rate were analyzed using a Cox proportional hazards model. A *p*-value of <0.05 was considered statistically significant.

### 2.5. AI Tools Statement

ChatGPT (OpenAI, GPT-5) was used for English language editing of the manuscript. No AI tools were used for data analysis, content generation, or interpretation of results.

## 3. Results

### 3.1. Baseline Characteristics

A total of 298 eyes from 221 patients were analyzed. Of these, 133 patients (162 eyes) underwent trabeculectomy, and 110 patients (136 eyes) underwent AGV implantation (Table 1). The mean age was significantly higher in the AGV group (67.8 ± 12 years) than in the trabeculectomy group (65.0 ± 12 years). The mean preoperative IOP was also significantly higher in the AGV group (27.1 ± 11 mmHg) compared to that in the trabeculectomy group (21.0 ± 9.9 mmHg; *p* < 0.001, Mann–Whitney U test). In contrast, no significant difference was observed between the two groups in terms of the preoperative anti-glaucomatous medication score. The distribution of FP agonist use was also comparable between the groups. However, there were significant differences in the distribution of glaucoma types between the two groups, and the number of prior glaucoma surgeries was significantly higher in the AGV group.

### 3.2. Baseline Characteristics Stratified by the Type of FP Agonist Used: The High-PAP-Potential Group and the Low-PAP-Potential Group

Patients were divided into two subgroups based on the type of FP agonist used preoperatively: a high-PAP-potential group (bimatoprost or travoprost) and a low-PAP-potential group (latanoprost or tafluprost). In the trabeculectomy group, 75 and 81 eyes were in the high- and low-PAP-potential groups, respectively. In the AGV group, 74 and 52 eyes were in the high- and low-PAP-potential groups, respectively. The baseline characteristics of patients are summarized in Table 2.

There were no significant differences in the distribution of glaucoma types or the number of eyes that received prior glaucoma surgeries between the high- and low-PAP-potential groups in either surgical group. Preoperative IOP was also comparable between the high- and low-PAP-potential groups within each surgical group.

However, in the trabeculectomy group, the preoperative anti-glaucomatous medication scores differed significantly between the high- and low-PAP-potential groups, with a higher score in the high-PAP-potential group (5.07 ± 1.1) relative to the low-PAP-potential group (4.49 ± 1.1; *p* < 0.001, Mann–Whitney U test).

### 3.3. Intraocular Pressure

**Figure 1:**
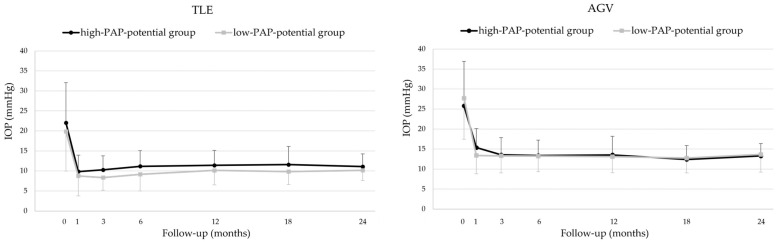
Postoperative intraocular pressure changes in groups stratified by the PAP-inducing potential of prostanoid FP receptor agonists. (**Left**): TLE group; (**Right**): AGV group. Eyes were divided into a high-PAP-potential group (bimatoprost or travoprost) and a low-PAP-potential group (latanoprost or tafluprost). In both surgical groups, IOP significantly decreased from the baseline at all follow-up points in both the high- and low-PAP-potential groups (Friedman test, *p* ≤ 0.001 at all time points). No significant differences in postoperative IOP were observed between the high- and low-PAP-potential groups in either the TLE or AGV groups (GEE analysis, all *p* > 0.4). AGV, Ahmed glaucoma valve; GEE, generalized estimating equation; IOP, intraocular pressure; PAP, prostaglandin-associated periorbitopathy; TLE, trabeculectomy.

Changes in IOP stratified by the PAP-inducing potential of FP agonists are shown in Figure 1. In the trabeculectomy group, both the high-PAP-potential (bimatoprost or travoprost) and low-PAP-potential (latanoprost or tafluprost) groups showed significant postoperative reductions in IOP at all follow-up points compared to the baseline (Friedman test, all *p* < 0.001). In the low-PAP-potential group, the IOP reduction was significant at all time points, with *p* = 0.001 at 1.5 years and *p* < 0.001 at all other time points.

In the AGV group, both the high- and low-PAP-potential groups also exhibited significant postoperative IOP reductions at all time points compared to the baseline (Friedman test, all *p* < 0.001).

When comparing the postoperative IOP course between the high- and low-PAP-potential groups, no significant differences were observed in either the trabeculectomy or AGV groups (GEE analysis, all *p* > 0.4). There were no significant differences in preoperative or postoperative IOP between the high- and low-PAP-potential groups in either the trabeculectomy or AGV groups (Table 3).

### 3.4. Anti-Glaucomatous Medication Scores

**Figure 2:**
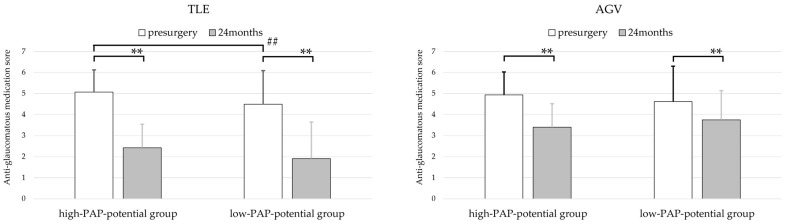
Preoperative (white bar) and 24-month postoperative (gray bar) anti-glaucomatous medication scores stratified by the PAP-inducing potential of prostanoid FP receptor agonists. (**Left**): TLE group; (**Right**): AGV group. ** *p* < 0.01 (Wilcoxon signed-rank test), ^##^ *p* < 0.01 (Mann–Whitney U test). Eyes were divided into a high-PAP-potential group (bimatoprost or travoprost) and a low-PAP-potential group (latanoprost or tafluprost). In both surgical groups, anti-glaucomatous medication scores significantly reduced from the baseline at 24 months postoperatively (Wilcoxon signed-rank test: TLE, *p* < 0.001 for both the high- and low-PAP-potential groups; AGV, *p* < 0.001 for high-PAP-potential group and *p* = 0.006 for low-PAP-potential group). The mean preoperative medication score in the TLE group was significantly higher in the high-PAP-potential group than in the low-PAP-potential group (Mann–Whitney U test, *p* < 0.001). Error bars represent standard deviations. AGV, Ahmed glaucoma valve; PAP, prostaglandin-associated periorbitopathy; TLE, trabeculectomy.

Anti-glaucomatous medication scores at baseline and at 24 months postoperatively were compared between the high-PAP-potential of FP agonists (bimatoprost or travoprost) and the low-PAP-potential of FP agonists (latanoprost or tafluprost) for both surgical procedures (Figure 2). In the trabeculectomy group, both the high- and low-PAP-potential groups showed significant reductions in medication scores at 24 months compared to the baseline (Wilcoxon signed-rank test, both *p* < 0.001). The mean preoperative medication score was significantly higher in the high-PAP-potential group (5.07 ± 1.1) than in the low-PAP-potential group (4.49 ± 1.1; Mann–Whitney U test, *p* < 0.001). However, at 24 months postoperatively, there was no significant difference between the groups (Mann–Whitney U test, *p* = 0.103).

In the AGV group, both the high- and low-PAP-potential groups also showed significant postoperative reductions in medication scores (Wilcoxon signed-rank test: high-PAP-potential group, *p* < 0.001; low-PAP-potential group, *p* = 0.006). There were no significant differences between the high- and low-PAP-potential groups in terms of either the preoperative scores (Mann–Whitney U test, *p* = 0.079) or the scores at 24 months (Mann–Whitney U test, *p* = 0.460).

### 3.5. Two-Year Cumulative Survival Rates and Factors Associated with Surgical Failure in Regard to Trabeculectomy

**Figure 3:**
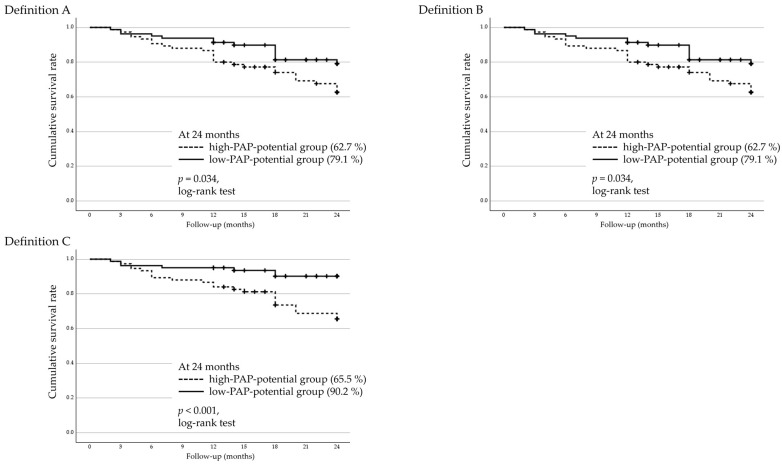
Kaplan–Meier survival curves for the trabeculectomy group stratified by the PAP-inducing potential of prostanoid FP receptor agonists. Eyes were divided into a high-PAP-potential group (bimatoprost or travoprost) and a low-PAP-potential group (latanoprost or tafluprost). Surgical failure was defined using three IOP–based criteria: Definition A, IOP > 21 mmHg or <20% reduction from the baseline; Definition B, IOP > 17 mmHg or <20% reduction; and Definition C, IOP > 14 mmHg. All criteria (Definitions (**A**)–(**C**)) were required to be met on two consecutive follow-up visits after 3 months postoperatively. The high-PAP-potential group showed significantly lower cumulative survival rates than the low-PAP-potential group under all three definitions (log-rank test). IOP, intraocular pressure; PAP, prostaglandin-associated periorbitopathy.

Two-year cumulative surgical survival rates following trabeculectomy were analyzed using Kaplan–Meier survival analysis and stratified by the PAP-inducing potential of FP agonists (Figure 3). The high-PAP-potential group (bimatoprost or travoprost) showed significantly lower survival rates than the low-PAP-potential group (latanoprost or tafluprost) under all three definitions (log-rank test): Definition A, *p* = 0.034; Definition B, *p* = 0.034; and Definition C, *p* < 0.001.

Factors associated with surgical failure pertaining to trabeculectomy were assessed using a Cox proportional hazards model for all three definitions (Table 4). The preoperative use of high-PAP-potential FP agonists was identified as a significant risk factor for surgical failure under all three definitions. Additionally, higher preoperative IOP was also a significant risk factor under Definition C.

### 3.6. Two-Year Cumulative Survival Rates and Factors Associated with Surgical Failure in AGV Implantation

**Figure 4:**
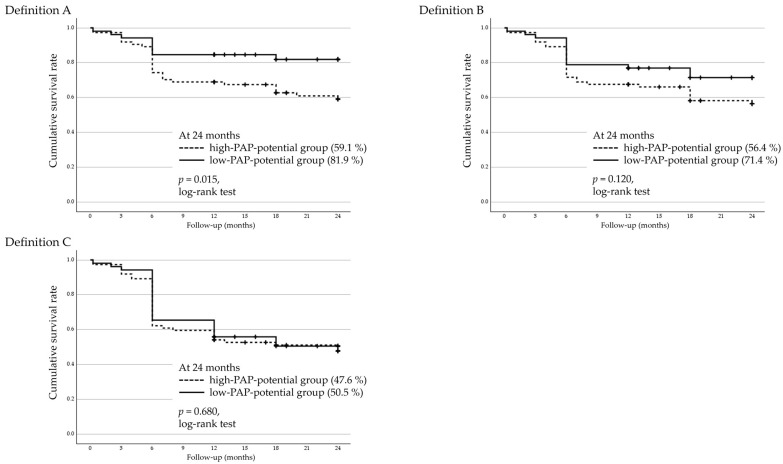
Kaplan–Meier survival curves for the AGV group stratified by the PAP-inducing potential of prostanoid FP receptor agonists. Eyes were divided into a high-PAP-potential group (bimatoprost or travoprost) and a low-PAP-potential group (latanoprost or tafluprost). Surgical failure was defined using three IOP–based criteria: Definition A, IOP > 21 mmHg or <20% reduction from the baseline; Definition B, IOP > 17 mmHg or <20% reduction; and Definition C, IOP > 14 mmHg. All criteria (Definitions (**A**)–(**C**)) were required to be satisfied on two consecutive follow-up visits after 3 months postoperatively. The high-PAP-potential group exhibited significantly lower cumulative survival rates than the low-PAP-potential group under Definition A (log-rank test, *p* = 0.015), whereas no significant differences were observed under Definitions B and C. AGV, Ahmed glaucoma valve; IOP, intraocular pressure; PAP, prostaglandin-associated periorbitopathy.

The two-year cumulative surgical survival rates following AGV implantation were analyzed using Kaplan–Meier survival analysis and stratified by the PAP-inducing potential of FP agonists (Figure 4). A significant difference was observed under Definition A, with the high-PAP-potential group (bimatoprost or travoprost) showing a lower survival rate than the low-PAP-potential group (latanoprost or tafluprost) (log-rank test, *p* = 0.015). However, no significant differences were found under Definitions B (log-rank test, *p* = 0.112) and C (log-rank test, *p* = 0.680).

Factors associated with surgical failure in AGV implantation were assessed using a Cox proportional hazards model for all three definitions (Table 5). The preoperative use of high-PAP-potential FP agonists was a significant risk factor only under Definition A. Under Definition C, higher preoperative IOP was significantly associated with surgical failure.

## 4. Discussion

FP agonists are widely used as first-line treatments for glaucoma, but they differ in their propensity to induce periorbital changes, such as PAP including DUES [1]. Bimatoprost and travoprost have been reported to promote eyelid stiffening and orbital fat atrophy by enhancing type I collagen expression and suppressing adipogenesis [2,3]. These histological changes underlie the development of PAP, which has been reported in 50–93% of patients treated with these agents [4,5,6,7]. In contrast, latanoprost and tafluprost are associated with a lower risk of such adverse effects. Although latanoprost also exhibits lipolytic and fibrotic activity [2,3], its limited accumulation in orbital tissues is thought to contribute to the lower reported incidence of DUES (6–41%) [5,6,8,9,10,19]. Tafluprost, a highly selective FP agonist with lower cytotoxicity and inflammatory potential [20,21], has been associated with low DUES rates of only 14–19% [5,11,12].

In this study, we stratified preoperative FP agonists into high-PAP-potential (bimatoprost and travoprost) and low-PAP-potential (latanoprost and tafluprost) categories based on their known propensity to cause PAP. We then compared the surgical outcomes of trabeculectomy and AGV implantation between these groups.

In the trabeculectomy group, the high-PAP-potential group exhibited significantly lower cumulative survival across all definitions of surgical failure. Cox proportional hazards analysis identified the preoperative use of high-PAP-potential FP agonists as a consistent predictor of surgical failure. Among the other tested factors, only higher preoperative IOP was significantly associated with surgical failure under Definition C, whereas glaucoma type, prior glaucoma surgery, and preoperative anti-glaucomatous medication score showed no significant associations. These findings suggest that PAP-associated changes—including eyelid stiffness, fibrosis, and fat atrophy—may compromise the formation and long-term viability of filtering blebs. Although this study did not include direct clinical imaging assessment of PAP or DUES, the observed outcome differences are consistent with previous reports highlighting the detrimental impact of PAP on trabeculectomy [13,14].

In the AGV group, a significant difference between the high- and low-PAP-potential groups was observed only under the lenient Definition A, while no significant differences were found under the stricter Definitions B and C. The Kaplan–Meier curves demonstrated a noticeable drop in survival rates around six months postoperatively across all groups. This timing coincides with the known hypertensive phase, which occurs in 56–82% of eyes within 3 to 6 months after AGV surgery and is associated with worse long-term outcomes [22,23,24]. The early postoperative decline in survival rates may indicate that the hypertensive phase had a greater impact on surgical outcomes, particularly for eyes with pre-existing PAP-associated changes, which were more common in the high-PAP-potential group. Definition A, being more permissive in terms of IOP criteria, may have been more sensitive in capturing these early failures.

Long-term exposure to bimatoprost or travoprost may result in advanced periorbital fibrosis and fat atrophy, potentially contributing to fibrous encapsulation of the AGV endplate and unstable postoperative IOP control during the hypertensive phase. In contrast, eyes treated with low-PAP-potential agents (latanoprost or tafluprost) may have had less pre-existing fibrosis, enabling them to maintain acceptable IOP control under a more permissive failure definition. However, under stricter criteria, the results did not reach statistical significance (Definition B: HR = 0.597, 95% CI 0.315–1.13, *p* = 0.120; Definition C: HR = 0.834, 95% CI 0.500–1.392, *p* = 0.487). The lack of significance under Definition B was most likely due to the limited number of events in the AGV subgroup, whereas under Definition C, the stringent cutoff (>14 mmHg) markedly reduced discriminatory ability, with approximately half of the cases classified as failures regardless of medication history. Although this threshold may indeed be overly demanding for tube shunt procedures that generally aim for relatively higher target pressures, we adopted it in line with previous landmark studies, such as the TVT and PTVT trials [16,17], to ensure comparability with prior research. Among the other tested factors, only higher preoperative IOP was significantly associated with surgical failure under Definition C, whereas glaucoma type, prior glaucoma surgery, and preoperative anti-glaucomatous medication score showed no significant associations. Although postoperative medication scores decreased significantly after AGV implantation, the absolute magnitude of this reduction was modest (approximately one medication), and thus its clinical significance should be interpreted with caution. These observations suggest that, while the influence of PAP is less prominent in AGV implantation compared to trabeculectomy, it should still be considered, particularly in the early postoperative period and for patients with a history of high-PAP-potential FP agonist use.

In trabeculectomy, bleb morphology is influenced by anterior periorbital tissues, and the SU-PAP grading system may be useful for predicting surgical outcomes [14]. In contrast, AGV implantation results in bleb formation approximately 8–10 mm posterior to the limbus. This deeper and more posterior anatomical configuration likely reduces the mechanical influence of PAP-related changes such as eyelid stiffness or orbital fibrosis, and thus the effects of PAP on AGV outcomes may be minimal. Although Harano et al. found no association between SU-PAP grades and AGV outcomes [18], this may have been due to the system’s reliance on external periorbital appearance, which may not fully reflect the condition of deeper orbital tissues surrounding the AGV bleb. Therefore, when evaluating indications for AGV and predicting surgical outcomes, a comprehensive assessment—including the severity of PAP and particularly the history of high-PAP-inducing FP agonist use—may be necessary.

This study has several limitations. First, as a retrospective study, direct clinical imaging assessment of PAP, including DUES, was not possible due to insufficient documentation in most medical records. Second, the classification into high- and low-PAP-potential groups was based solely on the final preoperative medication, without accounting for prior exposure, drug switching, or cumulative duration of use. Third, postoperative bleb morphology or anterior segment optical coherence tomography data were not available, which precluded analysis of their correlation with preoperative medication history. Fourth, because AGV is generally reserved for refractory cases, baseline disease severity and prior surgical history may have influenced the outcomes. Fifth, the relatively small sample size, particularly in the AGV group, may limit the applicability of our findings. Finally, as this is a single-center study, the results may not be fully generalizable to other populations or clinical settings.

## 5. Conclusions

This study demonstrates that preoperative use of high-PAP-inducing-potential agents was significantly associated with poorer surgical outcomes of trabeculectomy, while the impact was more limited in regard to AGV implantation. These findings underscore the importance of considering not only observable signs of PAP but also the patient’s medication history when selecting a surgical approach for glaucoma. Future prospective studies with larger sample sizes, longer follow-up periods, and direct assessment of PAP including DUES are warranted to further elucidate these associations and support evidence-based surgical decision-making.

## Figures and Tables

**Table 1:** Baseline characteristics of patients who underwent trabeculectomy and Ahmed glaucoma valve implantation.

		TLE *n* = 162 Eyes	AGV *n* = 136 Eyes	*p*
Gender (*n*)	Male	88	88	0.069 ^a^
	Female	74	48	
Age (years)		65.0 ± 12 (41~83)	67.8 ± 12 (31~90)	**0.002** ^b^
Preoperative IOP (mmHg)		21.0 ± 9.9 (9.0~58)	27.1 ± 11 (10~70)	**<0.001** ^b^
Preoperative anti-glaucomatous medication score		4.70 ± 1.2	4.68 ± 1.3	0.823 ^b^
Preoperative prostanoid FP receptor agonist (eyes)				0.214 ^a^
bimatoprost		62	56	
latanoprost		42	29	
tafluprost		39	23	
travoprost		13	18	
FP (-)		6	10	
Preoperative BCVA (logMAR)		0.174 ± 0.34 (−0.177~1.70)	0.446 ± 0.52 (−0.176~2.00)	**0.001** ^b^
Preoperative MD (dB)		−15.5 ± 7.4 (−0.290~−32.7) (*n* = 129)	−13.7 ± 7.2 (−0.790~−32.9) (*n* = 77)	0.074 ^b^
Lens status (eyes)	Phakic	98	69	**<0.001** ^a^
	IOL	64	67	
Glaucoma type (eyes)	POAG/NTG	97	61	**<0.001** ^a^
	PXG	21	16	
	Uveitis	29	15	
	NVG	3	14	
	others	12	30	
Prior glaucoma surgery (eyes)		65	85	**<0.001** ^a^

Values are presented as means ± standard deviations or counts. Statistical comparisons were performed using the Mann–Whitney U test (continuous variables) and Pearson’s χ^2^ test (categorical variables). Bold values indicate statistical significance (*p* < 0.05). a: the Pearson’s χ^2^ test, b: the Mann–Whitney U test. AGV, Ahmed glaucoma valve; BCVA, best-corrected visual acuity; dB, decibels; IOP, intraocular pressure; IOL, intraocular lens; MAR, minimum angle of resolution; MD, mean deviation; NTG, normal tension glaucoma; NVG, neovascular glaucoma; PXG, pseudoexfoliation glaucoma; POAG, primary open-angle glaucoma; TLE, trabeculectomy.

**Table 2:** Baseline characteristics stratified by the PAP-inducing potential of prostanoid FP receptor agonists: high-PAP-potential group (bimatoprost or travoprost) and low-PAP-potential group (latanoprost or tafluprost).

	**TLE**			**AGV**		
	**High-PAP-** **Potential** ***n* = 75 Eyes**	**Low-PAP-** **Potential** ***n* = 81 Eyes**	*p*	**High-PAP-** **Potential** ***n* = 74 Eyes**	**Low-PAP-** **Potential** ***n* = 52 Eyes**	*p*
Glaucoma type (eyes)			0.788 ^a^			0.305 ^a^
POAG	26	28		36	18	
NTG	19	23		2	3	
PXG	9	12		9	7	
Uveitis	9	16		6	8	
NVG	3	0		7	5	
Others	9	2		14	11	
Preoperative IOP (mmHg)	22.0 ± 10	19.8 ± 9.9	0.102 ^b^	25.8 ± 11	27.7 ± 10	0.105 ^b^
Preoperative anti-glaucomatous medication score	5.07 ± 1.1	4.49 ± 1.1	**<0.001** ^b^	4.93 ± 1.1	4.62 ± 1.1	0.079 ^b^
Prior glaucoma surgery (eyes) *			0.258 ^a^			0.695 ^a^
none	39	55		30	21	
SLT	20	15		26	14	
MP-CPC, CPC	1	1		5	4	
TLO	11	11		8	10	
TLE	12	6		25	19	
Tube shunt	2	2		6	2	

Comparisons between groups were performed using the Mann–Whitney U test for continuous variables and the Pearson’s χ^2^ test for categorical variables. Bold values indicate statistical significance (*p* < 0.05). a: the Pearson’s χ^2^ test, b: Mann–Whitney test. *: Prior glaucoma surgery includes overlapping counts when different procedures were performed on the same eye. AGV, Ahmed glaucoma valve; CPC, continuous-wave cyclophotocoagulation; IOP, intraocular pressure; MP-CPC, micropulse laser cyclophotocoagulation; NTG, normal tension glaucoma; NVG, neovascular glaucoma; PAP, prostaglandin-associated periorbitopathy; PXG, pseudoexfoliation glaucoma; POAG, primary open angle glaucoma; SLT, selective laser trabeculoplasty; TLE, trabeculectomy; TLO, trabeculotomy.

**Table 3:** Comparison of preoperative and 24-month postoperative intraocular pressure between groups stratified by the PAP-inducing potential of FP receptor agonists.

		High-PAP-Potential Group	Low-PAP-Potential Group	*p*
TLE	Pre-surgery IOP (mmHg)	22.0 ± 10	19.8 ± 9.9	0.102
	24 months IOP (mmHg)	11.1 ± 3.2	10.2 ± 2.5	0.197
AGV	Pre-surgery IOP (mmHg)	25.8 ± 11	27.7 ± 10	0.105
	24 months IOP (mmHg)	13.3 ± 3.1	13.6 ± 4.4	0.985

Values are presented as means ± standard deviations. Comparisons were performed using the Mann–Whitney U test. High-PAP-potential group: bimatoprost or travoprost; low-PAP-potential group: latanoprost or tafluprost. AGV, Ahmed glaucoma valve; IOP, intraocular pressure; PAP, prostaglandin-associated periorbitopathy; TLE, trabeculectomy.

**Table 4:** Cox proportional hazards analysis of factors associated with surgical failure regarding trabeculectomy under Definitions A, B, and C.

	Definition A		Definition B		Definition C	
	Hazard Ratio (95% CI)	*p*	Hazard Ratio (95% CI)	*p*	Hazard Ratio (95% CI)	*p*
Glaucoma type	1.09 (0.926–1.28)	0.305	1.09 (0.926–1.28)	0.307	1.04 (0.881–1.23)	0.627
Prior glaucoma surgery (Yes/No)	0.588 (0.301–1.15)	0.121	0.589 (0.301–1.15)	0.121	0.495 (0.231–1.06)	0.071
Preoperative IOP (mmHg)	0.989 (0.953–1.03)	0.556	0.989 (0.953–1.03)	0.559	1.04 (1.01–1.07)	**0.005**
Preoperative anti-glaucomatous medication score	1.07 (0.802–1.41)	0.664	1.06 (0.801–1.41)	0.666	1.08 (0.782–1.50)	0.632
FP agent (latanoprost or tafluprost/ bimatoprost or travoprost)	0.473 (0.242–0.923)	**0.028**	0.473 (0.243–0.923)	**0.028**	0.271 (0.115–0.636)	**0.003**

Hazard ratios and 95% confidence intervals (CIs) are shown. Bold values indicate statistical significance (*p* < 0.05). IOP, intraocular pressure.

**Table 5:** Cox proportional hazards analysis of factors associated with surgical failure in AGV implantation under Definitions A, B, and C.

	Definition A		Definition B		Definition C	
	Hazard Ratio (95% CI)	*p*	Hazard Ratio (95% CI)	*p*	Hazard Ratio (95% CI)	*p*
Glaucoma type	1.02 (0.895–1.17)	0.755	0.990 (0.877–1.12)	0.869	1.01 (0.906–1.11)	0.931
Prior glaucoma surgery (Yes/No)	0.592 (0.310–1.13)	0.111	0.625 (0.346–1.13)	0.120	0.729 (0.440–1.21)	0.220
Preoperative IOP (mmHg)	1.00 (0.967–1.03)	0.977	1.01 (0.983–1.04)	0.443	1.03 (1.00–1.05)	**0.026**
Preoperative anti-glaucomatous medication score	0.898 (0.686–1.18)	0.433	0.834 (0.652–1.07)	0.148	1.02 (0.815–1.27)	0.887
FP agent (latanoprost or tafluprost/ bimatoprost or travoprost)	0.402 (0.189–0.856)	**0.018**	0.597 (0.315–1.13)	0.120	0.834 (0.500–1.392)	0.487

Hazard ratios and 95% confidence intervals (CIs) are shown. Bold values indicate statistical significance (*p* < 0.05). IOP, intraocular pressure.

## Data Availability

Researchers can contact Masayo Kimura, (kimuram030301@yahoo.co.jp), to obtain details of the protocol and results.

## Abbreviations

The following abbreviations are used in this manuscript:

AGV	Ahmed glaucoma valve
ANOVA	analysis of variance
BCVA	best-corrected visual acuity
BGI	Baerveldt glaucoma implant
CI	confidence interval
CPC	cyclophotocoagulation
dB	decibels
DUES	deepening of upper-eyelid sulcus
GEE	Generalized estimating equation
IOL	intraocular lens
IOP	intraocular pressure
MAR	minimum angle of resolution
MD	mean deviation
MP-CPC	micropulse laser cyclophotocoagulation
NTG	normal tension glaucoma
NVG	neovascular glaucoma
PAP	prostaglandin-associated periorbitopathy
PXG	pseudoexfoliation glaucoma
POAG	primary open-angle glaucoma
PTVT	Primary Tube Versus Trabeculectomy
SLT	selective laser trabeculoplasty
SU	Shimane University
TLE	trabeculectomy
TLO	trabeculotomy
TVT	Tube Versus Trabeculectomy
